# Reversed Priming Effects May Be Driven by Misperception Rather than Subliminal Processing

**DOI:** 10.3389/fpsyg.2016.00198

**Published:** 2016-02-19

**Authors:** Anders Sand

**Affiliations:** Gösta Ekmans Laboratory, Department of Psychology, Stockholm UniversityStockholm, Sweden

**Keywords:** subliminal priming, unconscious processing, perception, stimulus strength, signal detection theory, trial-based analysis

## Abstract

A new paradigm for investigating whether a cognitive process is independent of perception was recently suggested. In the paradigm, primes are shown at an intermediate signal strength that leads to trial-to-trial and inter-individual variability in prime perception. Here, I used this paradigm and an objective measure of perception to assess the influence of prime identification responses on Stroop priming. I found that sensory states producing correct and incorrect prime identification responses were also associated with qualitatively different priming effects. Incorrect prime identification responses were associated with reversed priming effects but in contrast to previous studies, I interpret this to result from the (mis-)perception of primes rather than from a subliminal process. Furthermore, the intermediate signal strength also produced inter-individual variability in prime perception that strongly influenced priming effects: only participants who *on average* perceived the primes were Stroop primed. I discuss how this new paradigm, with a wide range of *d′* values, is more appropriate when regression analysis on inter-individual identification performance is used to investigate perception-dependent processing. The results of this study, in line with previous results, suggest that drawing conclusions about subliminal processes based on data averaged over individuals may be unwarranted.

## Introduction

A question with long-standing popularity among psychologists is to what extent our cognitive systems process stimuli that we do not perceive.^1^ Answering this question may reveal both whether and how unconscious information influences our decisions (Newell and Shanks, 2014) and help us understand the role of consciousness (Koch, 2004). Although there are many claims of high-level processing of unperceived stimuli, the question remains unresolved (e.g., Merikle, 2001; Erdelyi, 2004; Holender and Duscherer, 2004; Kanai et al., 2006; Van den Bussche et al., 2009; Finkbeiner and Coltheart, 2014; Newell and Shanks, 2014).

To test whether a cognitive process is independent of perception, researchers often compare two measures of stimulus processing at a signal intensity meant to render perception impossible. One measure is a direct measure of stimulus perception, typically a prime identification task with performance measured in terms of the signal detection theory sensitivity measure *d′* (Green and Swets, 2000; Macmillan and Creelman, 2005). The other measure is an indirect measure of stimulus processing, typically a priming effect on reaction times in an indirect task. If, on a mean (group) level, the direct measure suggests that the stimulus was not perceived while the indirect measure suggests that the stimulus influenced behavior, this is often interpreted as suggesting that perception and the implied cognitive process are dissociated (Reingold and Merikle, 1988).

This standard paradigm is still widely used (e.g., Finkbeiner, 2011; Muscarella et al., 2013; González-García et al., 2015; Schoeberl et al., 2015) but has been criticized as not strongly supporting conclusions either for or against subliminal processing. To conclude that a cognitive process is independent of perception, researchers must first support the claim that the stimulus was subliminal—not even slightly perceived—for all participants. Researchers often try to support this claim using a critical signal intensity that restricts the range of *d′* to chance levels and by applying a null-hypothesis significance test to demonstrate that mean performance was not different from chance. This analytical approach has been criticized because of lack of power in the test (e.g., Rouder et al., 2007; Finkbeiner and Coltheart, 2014), because of the possible underestimation of *d′* (e.g., Pratte and Rouder, 2009; Vermeiren and Cleeremans, 2012; Lin and Murray, 2014), and because a non-significant test cannot support the conclusion experimenters want to reach (e.g., Gallistel, 2009; Dienes, 2015; Sand and Nilsson, under review). On the other hand, to conclude that a cognitive process is dependent on perception, experimenters must support the claim that a lack of indirect processing was due to a lack of perception *per se* and not due to an insufficient signal intensity (Lau, 2009; Van den Bussche et al., 2013). As such, it can often be difficult to know what to conclude from this standard paradigm.

Van den Bussche et al. (2013) therefore recently suggested a new paradigm for testing whether a cognitive process is dependent on perception. Instead of testing mean outcomes at a critical signal intensity used to restrict *d′* to chance levels, they used an intermediate signal intensity and a trial-based approach to classify responses as conscious, uncertain, or unconscious based on self-reports. This approach relies on the signal detection theory concept that any signal intensity will generate variability in sensory states (Green and Swets, 2000; Macmillan and Creelman, 2005) and that a stimulus that is *on average* difficult to perceive may sometimes be perceived and sometimes not (see also Lutz and Thompson, 2003). Haase and Fisk (2015) empirically supported this notion by demonstrating that different self-reported sensory states corresponded to different *d′* outcomes. By comparing trials in which participants reported perceiving or not perceiving the prime, Van den Bussche et al. (2013) were able to investigate whether a cognitive process was dependent on perception without proving that the stimulus was subliminal on a mean (group) level and while keeping signal intensity constant in both perceived and not-perceived trials. Similar trial-based analysis has been used to investigate perception-dependent processing in the past (e.g., Kahan, 2000; Lamy et al., 2009; Ro et al., 2009; Goodhew et al., 2011; Haase and Fisk, 2011; Peremen and Lamy, 2014; Hesselmann et al., 2015).

Van den Bussche et al. (2013) investigated how trial-to-trial variability in perception influenced semantic processing (Stroop priming), a process previously suggested to be independent of perception (e.g., Marcel, 1983; Merikle, 2001; Kouider and Dehaene, 2007; Huckauf et al., 2008; Costello et al., 2009; Van den Bussche et al., 2009, 2013). In Van den Bussche et al. (2013) study, participants reported their awareness of a prime word (“I am certain that I saw the color word Red,” “I think I saw the color word Red, but I am not certain,” and “I did not see the color word”) and the authors found the self-reports to be strongly associated with the size of the priming effects. This finding is in line with the results of other studies using a similar trial-based approach based on self-reports (e.g., Lamy et al., 2009; Ro et al., 2009). Van den Bussche et al. (2013) also reported a statistically significant priming effect in the trials participants themselves reported to be unconscious (“I did not see the color word”; mean effect = 33 ms, 95% CI = 0.35–65.65 ms), indicating that Stroop priming can occur independent of perception (Van den Bussche et al., 2013).

However, as was also discussed by Van den Bussche et al. (2013), the validity of self-reports may be questioned (Eriksen, 1960; Cheesman and Merikle, 1984; Holender, 1986; Björkman et al., 1993; Wiens, 2007). Although introspective judgments of perception have face validity (Cleeremans, 2011), it is also clear that a self-reported lack of perception may not indicate an actual lack of perception but rather a lack of confidence (Holender, 1986; Björkman et al., 1993; Wiens, 2007). This may be especially problematic when self-reports are used in isolation to divide trials into conscious and unconscious bins (Schmidt, 2015). In the absence of a consensus on how to measure perception, it is therefore important to compare effects based on both types of measures. Here, the paradigm suggested by Van den Bussche et al. (2013) was used, but I follow other studies in dividing trials into different sensory states based on objective performance (e.g., Kahan, 2000; Lamy et al., 2009; Goodhew et al., 2011). I hypothesize that an intermediate signal strength will produce trial-to-trial variability in sensory states that in turn will produce correct and incorrect prime identification responses (plus measurement error in translating the sensory state to a response) and different priming effects. Based on previous studies, incorrect prime identification responses may be associated with either no priming (Lamy et al., 2009) or even reversed priming effects (Kahan, 2000; Goodhew et al., 2011).

As participants differ in sensory thresholds and thus prime perception given a fixed signal intensity (e.g., Dagenbach et al., 1989; Greenwald et al., 1995; Albrecht and Mattler, 2012; Haase and Fisk, 2015; Lin and Murray, 2015), I also wanted to test how inter-individual variability in prime perception influences priming effects. A strength of the paradigm developed by Van den Bussche et al. (2013), although not explicated by the authors, is that a signal intensity was used that did not restrict the range of identification performance to chance levels but rather allowed for natural variability between participants (in the study by Van den Bussche et al., 2013, the mean proportion of correct responses was 59% [chance 50%], standard deviation 15%-points, min 27% and max 89%; personal communication). This variability in prime perception enables regression analysis as an approach to test whether a process is dependent on perception, an analytical approach otherwise demonstrated to be unreliable (Dosher, 1998; Miller, 2000; Sand and Nilsson, under review). By allowing a natural inter-individual variability in prime perception, Haase and Fisk (2015) recently reported that such variability can indeed influence priming effects. Here, I investigated how inter-individual variability in prime perception influenced priming effects in both correct and incorrect prime identification trials.

The paradigm developed by Van den Bussche et al. (2013) was thus used to investigate two hypotheses: (1) given a constant signal strength, sensory states that produce correct or incorrect prime responses should also be associated with different priming effects, and (2) given a constant signal strength, inter-individual variability in perception should influence priming effects. Self-reports of prime perception were also measured to compare the present results with those of Van den Bussche et al. (2013). The aim of the current experiment was not to provide a perfect measure of prime perception on a trial level, but rather to demonstrate the possibility of variable priming effects from sensory states that produce correct and incorrect responses.

## Materials and Methods

### Participants

Fifty-three participants with different backgrounds participated in the experiment (one participant was excluded for not following instructions). There were 32 females and 21 males, all aged 18–28 years except for one 48-years-old participant. All participants had normal or corrected-to-normal vision. Like Van den Bussche et al. (2013), I piloted 21 other participants prior to the experiment to find inter-stimulus-intervals (ISIs) between the prime and mask that allowed for both trial and individual variability in perception. All participants were naïve to the purpose of the experiment, gave informed consent and were compensated with a cinema ticket. The research was conducted in accordance with the principles of the regional ethics board.

### Apparatus

Stimuli were shown at a distance of 0.5 m on a View Sonic P22f CRT monitor (1024 × 768 pixels; ViewSonic, Walnut, CA, USA) using Presentation 16.3 software (Neurobehavioral Systems, Berkeley, CA, USA). Viewing position and distance were stabilized with a chinrest. Stimulus presentation was synchronized with the screen refresh rate of 160 Hz. All stimuli were presented at the center of the screen against dark gray background.

### Stimuli

Each experimental trial started with a central fixation cross (width, 0.6° visual angle) shown between 500 and 900 ms. Following this, one of four different words denoting color was shown (referred to as primes; “red,” “blue,” “green,” and “yellow”^2^; 1°; 6-ms duration; shown in gray font). An ISI of 12.5, 25, or 100 ms separated the prime from a following backward mask consisting of six number signs (######; 2.8°; 140 ms). Then a red or blue colored square was shown (referred to as a target; 2°; 140 ms). Finally, a central question mark (1.5°) was shown until a response to the target was given. A 2 × 2 grid was then displayed showing the different prime alternatives. Four prime words were used to reduce the number of expected correct guesses to 25% and thus more clearly distinguish correctly perceived from correctly guessed trials.

### Procedure

Participants were first required to make a speeded response to the target, categorizing it as either “red” or “blue” using the “F” and “J” keys on the keyboard. After this, participants used the mouse to select which prime they had seen from the displayed grid (unspeeded four-alternative-forced-choice). After selecting their prime response, participants had to rest their fingers on the keyboard before initiating the next trial.

There were 144 trials at an ISI of 12.5 ms and 96 trials each at ISIs of 25 and 100 ms. As piloting had shown that the 12.5-ms ISI was difficult to perceive for many participants, a larger trial number was used for this ISI to enable trial-based analysis. Using 144 trials in all ISIs would have increased the experimental duration above 1 h. The 100-ms ISI was included because previous studies have suggested that intermixing easier ISIs may increase motivation in the prime identification task (Pratte and Rouder, 2009). For each ISI, 50% of the trials were congruent. The experiment was divided into six blocks separated by pauses between (each block consisting of 56 trials with an equal combination of ISIs and congruent trials pseudorandomly intermixed). After the experiment, the last 41 participants of the experiment were asked in a questionnaire to report how often they had perceived the primes (proportion of trials).

Participants had to dual-task in the experiment, responding to the target and then the prime in each trial. Although dual-tasking may have some impact on priming effects, previous studies using dual-tasking have found strong priming effects (e.g., Kahan, 2000; Lamy et al., 2009; Ro et al., 2009; Finkbeiner, 2011; Goodhew et al., 2011; Van den Bussche et al., 2013). Dual-tasking can be difficult for the participant, however, so the experiment started with two training phases. In the first phase, participants only had to respond to the target color (no prime was shown during this training session) and were trained on the response mappings in 56 trials. Three participants were not confident in their responses and were allowed a second training session of 56 trials. In the second phase, participants trained on the combined task of first responding to the target and then to the prime while maintaining a fast response to the target. In this second training phase, ISIs between the prime and mask were 61, 72, and 150 ms (38 trials with one third allotted to each ISI and one third being congruent). During this training phase, participants received verbal encouragement and instructions to help them identify the primes. In the training phases, participants also adapted to the average luminance of the display.

## Results

### Result Overview

**Table 1** summarizes the group-level results for the different ISIs.

**Table 1:** General results on a group level.

	Stroop priming (ms)		Error rate (%)		Prime identification (*d′*)	
ISI (ms)	Mean (SD)	95% CI	Mean (SD)	95% CI	Mean (SD)	95% CI
12.5	6.10 (31.56)	-2.69–14.88	0.67 (3.31)	-0.25–1.59	0.22 (0.27)	0.14–0.29
25	26.26 (56.74)	10.47–42.06	1.02 (2.88)	0.22–1.82	0.72 (0.59)	0.56–0.88
100	118.75 (66.90)	100.13–137.38	3.90 (3.95)	2.80–5.00	2.98 (1.67)	2.51–3.44
Across	39.21 (29.95)	30.87–47.55	5.60 (7.00)	3.65–7.55	0.89 (0.39)	0.78–0.99

*Stroop priming is the median difference (in ms) between responses to targets following incongruent primes minus responses to targets following congruent primes. Error rate is the mean difference (in %-points) between errors in responses to targets following incongruent primes minus errors in responses to targets following congruent primes. Across is the mean across the different ISIs. SD, Standard deviation; CI, confidence interval.*

#### Objective Perception

Mean prime identification across all ISIs was quite high, with the prime being correctly identified in 52% of the trials (*SD* = 12%-points; chance performance would be approximately 25%). Prime identification performance decreased with shorter ISIs but was, as hypothesized, quite variable at ISIs of both 12.5 and 25 ms. On a mean level, prime identification was above chance performance at all ISIs (**Table 1**).

**FIGURE 1:**
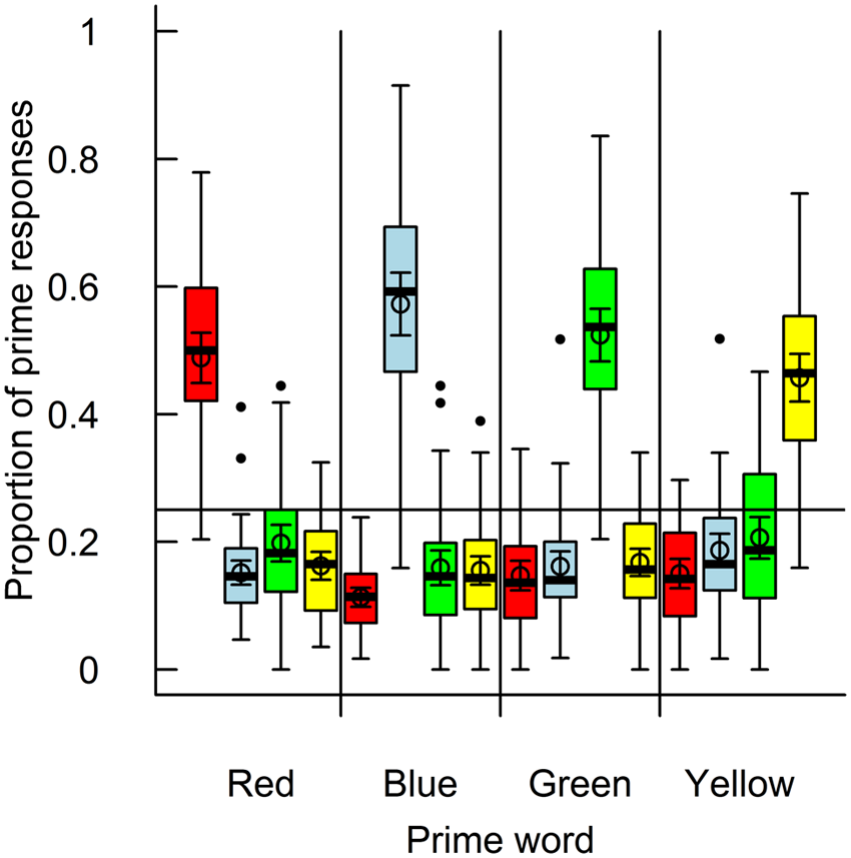
**Proportion of prime responses for each of the four prime words.** The prime word is shown on the *x*-axis and prime responses are illustrated as the color of the boxplots (in the same order). Mean and 95% confidence intervals are superimposed.

Because there were four prime words but only two targets, each prime was not equally likely to be shown in each trial. This means that participants were more likely to be correct if they guessed ‘red’ or ‘blue’ as their prime response compared to ‘yellow’ or ‘green.’ Control analyses were performed to test that this did not bias participants in their prime responses. In a first analysis, I examined how often participants responded that the prime was the same as the target in incongruent trials. Responding in this manner in 25% of the trials would suggest zero bias whereas a greater proportion would suggest bias. The proportion on a group level was 22% of the incongruent trials (*SD* = 11%-points). In a second analysis, I examined how often the different prime responses were used for each prime shown. This was done in order to see if there was a bias for picking ‘red’ or ‘blue’ (the possible target colors) over the other options. The result of this analysis is shown in **Figure 1**. As can be seen, there was no response bias for any of the primes. I also performed these analyses separately for participants who identified the primes better than chance and those who did not, no differences were found. As such, these analyses suggests little to no bias in the participants’ prime responses.

#### Self-Reported Perception

**FIGURE 2:**
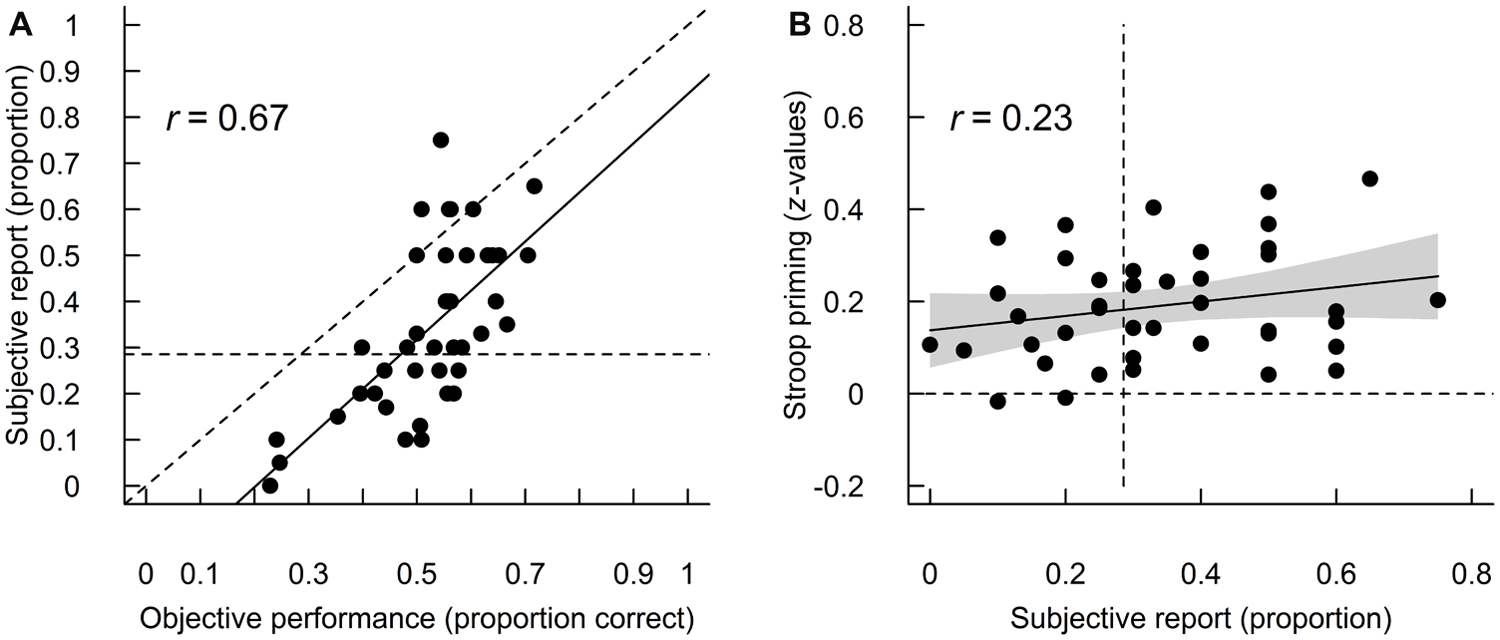
**(A)** Correlation between participants’ objective prime identification performance across all ISIs and self-reported proportion of perceived primes. Proportion correct rather than *d′* is shown here for comparison with self-reports. The straight line shows the fitted regression slope. For reference, the diagonal is shown as a dotted line and the proportion of primes is shown with an ISI of 100 ms as the horizontal dotted line. **(B)** Relationship between participants’ self-reported proportion of perceived primes and Stroop priming (as *z*-values) across all ISIs. The straight line shows the fitted linear regression slope and the gray polygon is its 95% confidence interval. For reference, the vertical dotted line is the proportion of primes shown with an ISI of 100 ms and the horizontal dotted line is zero priming.

Self-reports from the 41 participants who contributed with these, indicated that they perceived few primes (mean = 34% perceived primes, *SD* = 18%-points) in all trials. For reference, 29% of the primes were shown at an ISI of 100 ms, which were clearly visible based on objective performance. **Figure 2A** illustrates the relationship between self-reports and objective performance across all ISIs. Most, i.e., 88%, of the self-reports were below the diagonal, indicating that participants generally reported seeing fewer primes than their objective performance would suggest (in line with previous literature; Cheesman and Merikle, 1984; Lamy et al., 2009).

#### Stroop Priming

To analyze reaction times, all trials in which the target response was incorrect were excluded (8.1% of all trials). Following Van den Bussche et al. (2013), no trials were excluded based on response time (RT), to include more trials in the trial-based analysis. Additional analyses excluding trials slower than two standard deviations gave similar results. Participants were generally slower when responding to a target following an incongruent prime than to a target following a congruent prime, indicating a general Stroop effect (**Table 1**). When the primes were clearly seen (at 100-ms ISI), the Stroop effect was robust for almost all participants (62% had priming effects greater than 100 ms); the Stroop effect decreased at shorter ISIs. Two prime words (‘red’ and ‘blue’) were in conflict with a target response and the other two prime words (‘yellow’ and ‘green’) were not. A control analysis suggested that priming effects did not differ between conflicting and non-conflicting primes. Priming for conflicting primes across all ISIs was 44.20 ms (95% CI = 31.63–56.77 ms) and for non-conflicting primes 38.74 ms (95% CI = 30.35–47.12 ms).

#### Error Analyses

For comparison with the study by Van den Bussche et al. (2013) error rates for the different ISI were also analyzed. Error rates followed the same pattern as RT effects for the different ISIs (**Table 1**). No trial-based analysis on Error rates was performed.

### Trial-to-Trial Variability in Prime Perception

Our first hypothesis was that trial-to-trial variability in sensory state should influence priming effects. To test this, trials in the 12.5 and 25 ms ISI condition were divided according to whether or not the prime was correctly identified (71% of participants correctly identified over 90% of primes at 100-ms ISI, making similar analyses for that ISI less meaningful). This division led to a variable number of trials per condition for each participant; on a mean level, 28 trials (*SD* = 9) were included in each condition. Previous experiments have used different arbitrary cutoffs to exclude participants, for example, excluding participants with fewer than 4 or 15 trials in each condition (Haase and Fisk, 2011; Van den Bussche et al., 2013). However, excluding participants based on the number of correct or incorrect trials may introduce data interpretation problems (e.g., regression to the mean; Shanks and Berry, 2012; Sand and Nilsson, under review), so all participants were included in all analyses here. Additional analyses excluding participants based on different cutoffs gave similar results.

**Table 2** reports the mean of median RTs for correct and incorrect prime identification responses for the 12.5- and 25-ms ISIs. In line with our hypothesis and several previous studies, trial variability in prime perception did influence priming effects. On a group level, participants had quite large congruency effects at both 12.5- and 25-ms ISIs in the trials with correct prime identification responses. In comparison, participants had reversed congruency effects at both 12.5- and 25-ms ISIs in the trials with incorrect prime identification responses. In the omnibus ANOVA with the factors prime response, prime identification response, and ISI, the interaction between prime response and congruency was significant, i.e., *F*_1,51_ = 14.73, *p* < 0.001. The other significant effects were the main effect of congruency (*F*_1,51_ = 14.04, *p* < 0.001) and ISI (*F*_1,51_ = 10.10, *p* = 0.003). No other effects were statistically significant.

**Table 2:** Priming for correct and incorrect responses.

Prime response	ISI	Congruent, mean (SD)	Incongruent, mean (SD)	Congruency effect mean (95% CI)
Correct	12.5 ms	504.83 (142.43)	543.23 (211.61)	38.40 (2.68–74.13)
	25 ms	517.41 (175.47)	576.74 (209.14)	59.34 (34.60–84.07)
Incorrect	12.5 ms	524.69 (164.18)	515.13 (153.31)	-9.56 (-19.04– -0.09)
	25 ms	565.50 (229.61)	544.62 (214.86)	-20.88 (-44.12–2.37)

*Mean of the median RTs (in ms) as a function of prime identification response (correct and incorrect) and ISI for congruent and incongruent trials. Congruency effect is the median difference (in ms) between congruent and incongruent primes. SD, Standard deviation; CI, confidence interval.*

### Inter-Individual Variability in Prime Perception

Our second hypothesis was that inter-individual variability in perception should influence priming effects. Different regression analyses were therefore performed in which prime perception was used as the regressor and priming effects as the outcome variable. A correlation between the two variables would suggest that perception influence priming effects and a statistically significant intercept would suggest that priming occurred when *d′* was at zero (i.e., priming was subliminal; Greenwald et al., 1995). For the regression analyses, before calculating median RTs, the RTs were transformed to *z*-values to decrease between-subject variability.

First, I tested how the self-reported proportion of perceived primes was related to Stroop priming for the 41 participants who contributed with self-reports. **Figure 2B** illustrates this relationship. As the self-reported proportion of perceived primes was measured across ISIs, priming effects across ISIs were entered into this regression. The regression result suggested only a weak relationship between self-reports and priming (slope = 0.16, 95% CI = -0.05–0.37, *R*^2^ = 0.06), in line with previous reports (e.g., Cheesman and Merikle, 1984).

**FIGURE 3:**
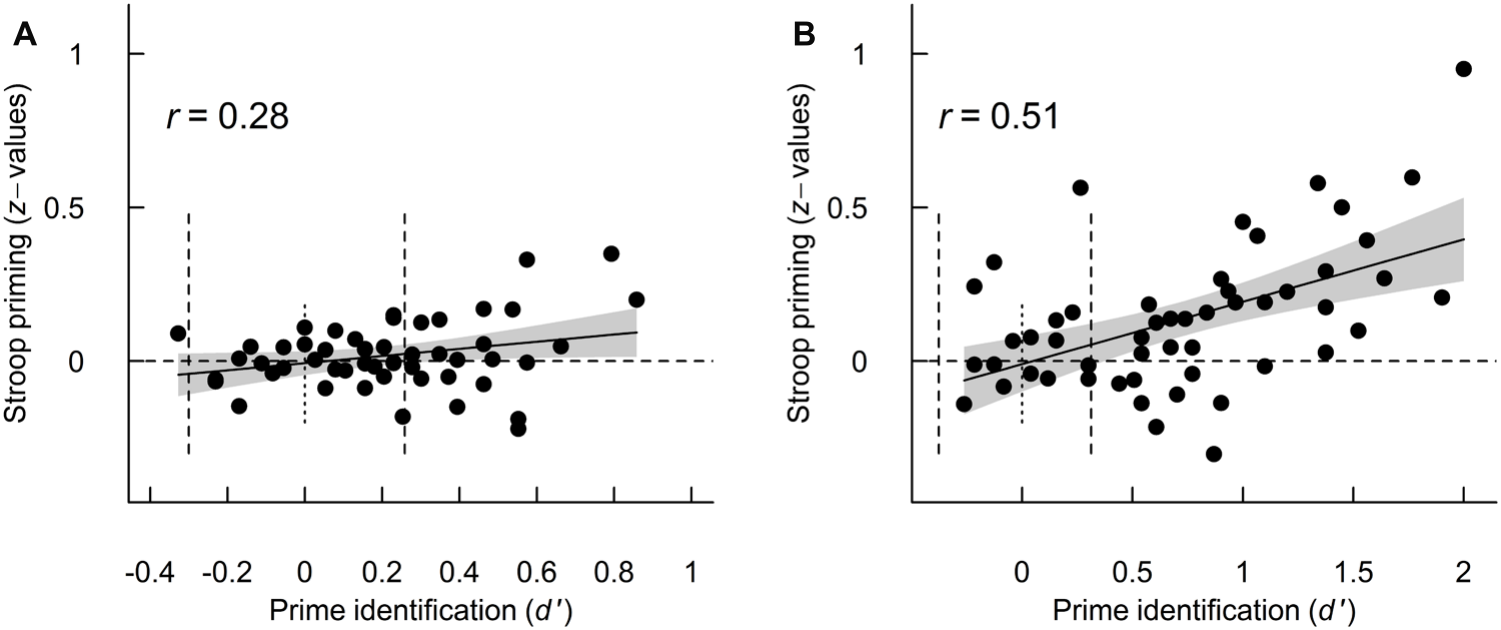
**Relationship between *d′* and Stroop priming (as *z*-values) for the 12.5-ms (A) and 25-ms ISIs (B).** In both figures, the straight line shows the fitted linear regression slope and the gray polygon is its 95% confidence interval. Note the difference in *x*-axis between the two figures. For reference, the vertical dotted lines show chance performance and the 95% performance levels based on the binomial distribution.

Next, I tested how inter-individual variability in *d′* was related to Stroop priming at 12.5- and 25-ms ISIs (across correct and incorrect prime responses). As mentioned above, inter-individual variability in prime perception was highly variable at both 12.5-and 25-ms ISIs (**Table 1**), with 60 and 31% of participants performing at levels that could be expected by chance (95% quantile of binomial distribution). **Figure 3** illustrates these relationships and **Table 3** summarizes the regression models. The regression results indicated a strong, positive relationship between *d′* and priming at the 25-ms ISI and a weaker, positive linear relationship at the 12.5-ms ISI. In general, robust priming effects were evident only for participants performing better than chance. The regression results did not suggest subliminal priming at either ISI (intercepts did not differ statistically significantly from zero). As such, at both an *on average* very-difficult-to-identify signal intensity and an *on average* less-difficult-to-identify signal intensity, individual variability in prime perception influenced priming effects and there was no indication of subliminal priming.

**Table 3:** Influence of individual variability on priming.

Prime response	ISI	Intercept (95% CI)	Slope (95% CI)	*R*^2^
Across	12.5 ms	-0.01 (-0.05–0.03)	0.12 (0.01–0.23)	0.08
	25 ms	-0.01 (-0.10–0.08)	0.20 (0.11–0.30)	0.26
Correct	12.5 ms	-0.01 (-0.14–0.12)	0.63 (0.25–1.01)	0.18
	25 ms	0.04 (-0.09–0.18)	0.34 (0.19–0.49)	0.30
Incorrect	12.5 ms	-0.01 (-0.06–0.04)	-0.15 (-0.30– -0.01)	0.08
	25 ms	0.04 (-0.09–0.17)	-0.17 (-0.31– -0.03)	0.11

*Regression models when Stroop priming (as z-value) was used as the outcome variable and prime identification (d′) as the regressor. Across are all trials for that ISI, collapsed across correct and incorrect prime identification response. CI, confidence interval.*

**FIGURE 4:**
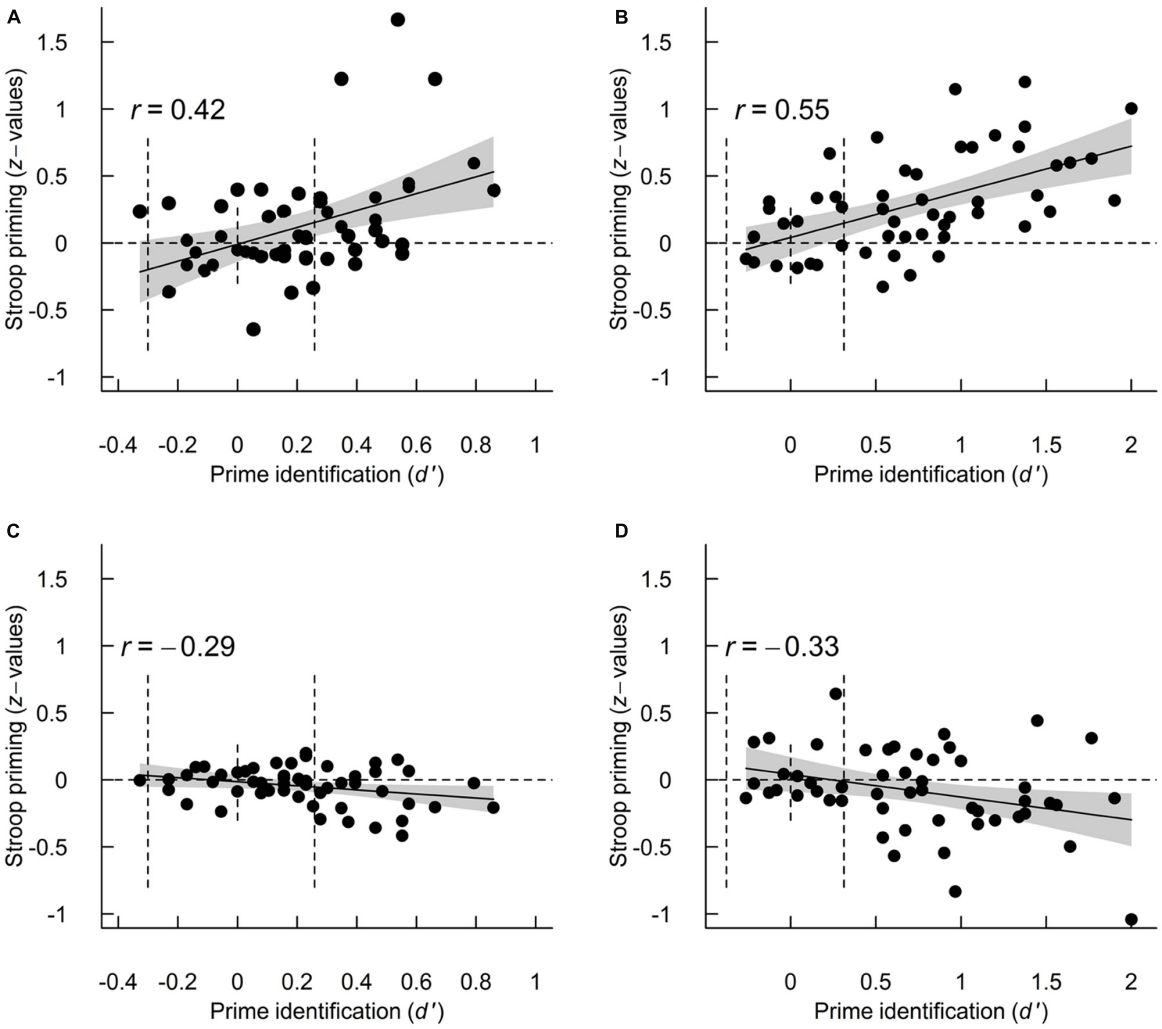
**Relationship between *d′* and Stroop priming (as *z*-values) for correct (A,B) and incorrect (C,D) prime identification responses for the 12.5-ms (A,C) and 25-ms ISIs (B,D).** In the upper panels, priming is calculated as correct incongruent – correct congruent, and in the lower panels as incorrect incongruent – incorrect congruent. In all panels, the straight line shows the fitted linear regression slope and the gray polygon is its 95% confidence interval. Note the difference in *x*-axis between the left and right panels. For reference, the vertical dotted lines show chance performance and the 95% performance levels based on the binomial distribution.

Finally, I tested how inter-individual variability in *d′* was related to stroop priming for correct and incorrect prime identification responses at the two ISIs. **Figure 4** illustrates the relationship for each of these four conditions and **Table 3** summarizes the regression models. For correct prime identification responses, the regression results indicated a strong, positive relationship between *d′* and priming effects at both ISIs. For incorrect prime identification responses, the regression results indicated a negative relationship between *d′* and priming effects at both ISIs. None of the regression models suggested any subliminal priming. Additional analyses excluding participants with absolute priming effects larger than a *z*-value of one gave similar results for all regression models.

## Discussion

With regard to the first hypothesis, I found trial-to-trial variability in perception to be strongly related to the priming effects of a difficult-to-perceive stimulus (**Table 2**), a finding in line with several previous studies (e.g., Kahan, 2000; Lamy et al., 2009; Ro et al., 2009; Goodhew et al., 2011; Van den Bussche et al., 2013). However, here priming differed qualitatively depending on the prime responses: correct prime identification responses were generally associated with Stroop priming whereas incorrect prime identification responses were generally associated with reversed priming effects (**Figure 4**). My interpretation (in line with Lutz and Thompson, 2003; Haase and Fisk, 2015) is that the signal strengths used here produced different sensory states, which in turn led to correct or incorrect prime responses and to qualitatively different priming effects.

Reversed priming effects for incorrect prime identification responses have been reported previously (e.g., Kahan, 2000; Goodhew et al., 2011). Previous studies proposed subliminal processing to account for the reversed priming effects: interpreting incorrect responses as indexing non-perceived stimuli and interpreting reversed priming as resulting from subliminal processing. Goodhew et al. (2011), for example, interpreted reversed priming as an effect of suppressing the response associated with the non-perceived stimulus, which in turn facilitates the opposite response (a negativity compatibility effect; Eimer and Schlaghecken, 1998). However, the present data do not indicate that the reversed priming (or the standard priming) was independent of perception, but rather that only participants who *on average* perceived the primes displayed reversed priming (**Figures 4C,D**). In contrast, I propose an explanation that does not require subliminal processing: incorrect prime identification responses may have indexed misperceived primes for participants who on average perceived the primes (they truly misperceived, e.g., “red” as “blue”). My interpretation of the reversed priming effect is that participants were primed by what they (mis-)perceived and not by subliminal processing of what was shown on the screen. That is, the misperception of the word ‘red’ had a tendency to prime the color red, even if the prime word ‘blue’ was actually shown on the screen. This explanation is consistent with the partial awareness hypothesis about consciousness (Kouider et al., 2010) and Kouider and Dupoux’s (2004) finding that partial awareness can drive priming effects. This interpretation can also be applied to previous studies in which (some) participants performed above chance (e.g., Kahan, 2000; Goodhew et al., 2011). Participants in the study by Goodhew et al. (2011), for example, produced correct prime identification responses in 72% of the trials (chance 50%) and thus may have in some of the incorrect prime response trials misperceived rather than not perceived the prime. Future studies that combine objective and subjective measures of prime perception in each trial may be able to lend support to one of these explanations.

With regard to the second hypothesis, inter-individual variability in prime perception strongly influenced the priming effects of a difficult-to-perceive stimulus (**Figures 3** and **4**). This indicates that participants who generally perceived the primes (based on *d′*) were primed by what they perceived or misperceived, whereas participants who generally did not perceive the primes were neither stroop or reversed primed by what little information they extracted from the primes. This finding is in line with the reports of Haase and Fisk (2015), who also used a stimulus setting that allowed for individual variability in prime perception but is in contrast with other reports that did not (e.g., Greenwald et al., 1995; Fisk and Haase, 2011; Schoeberl et al., 2015). Fisk and Haase (2011) interpreted their lack of a correlation between prime perception and priming as due to variable and small effect sizes, an explanation I agree with. Using regression analysis to investigate whether a process is independent of consciousness has previously been criticized as measurement error, and a restricted range of prime identification performance makes the approach unreliable (Dosher, 1998; Miller, 2000; Sand and Nilsson, under review). In the paradigm used here, in comparison, participants’ *d′* was not restricted to chance-level performance but rather allowed to vary naturally (with a variability similar in size to previous reports; Dagenbach et al., 1989; Albrecht and Mattler, 2012; Van den Bussche et al., 2013; Haase and Fisk, 2015; Lin and Murray, 2015), which increases the chances of finding a correlation between prime perception and priming. Future studies that use a regression approach to test whether a process is independent of consciousness should therefore use a signal strength that gives rise to variability in prime identification performance.

Using an objective measure of perception I found no indication of subliminal Stroop priming (**Table 3**). This is in conflict with the result of Van den Bussche et al. (2013), who used a similar paradigm (see below for a discussion about differences in experimental parameters), but a subjective measure of perception and who reported subliminal Stroop priming. This difference may simply stem from the well-known fact that subjective measures are less strict than objective measures (Eriksen, 1960; Cheesman and Merikle, 1984; Holender, 1986; Björkman et al., 1993; Wiens, 2007). In a study by Lamy et al. (2009), for example, participants performed 59% correctly on prime identification (chance performance 25%) in the trials where they rated themselves as unaware. Participants in the present study also reported perceiving fewer primes than their objective performance would suggest (**Figure 2A**). Furthermore, in line with previous reports (e.g., Cheesman and Merikle, 1984), there was no strong relationship between the self-reported proportion of perceived primes and priming effects (**Figure 2B**). If I extrapolate from our self-report data, many trials at the 12.5- and 25-ms ISIs might have been unconscious based on self-reports and, in that sense, have given rise to unconscious processing.

Furthermore, the present data are also in conflict with several studies that used an objective measure of perception and that did report subliminal semantic priming (e.g., Finkbeiner, 2011; Muscarella et al., 2013). The lack of support for subliminal priming in the present study may be explained by the paradigm used. First, a lack of subliminal priming might generally stem from a too weak signal strength (as discussed above and in Van den Bussche et al., 2013). There are two arguments against this explanation in the present study: (i) In comparison to previous studies that have generally used signal strengths that were subliminal for most or all participants, the intermediate signal strength used here was supraliminal for many participants. As this study used a stronger signal strength than many previous studies, there are no *a priori* reasons to assume that there would be no subliminal priming. (ii) The prime did elicit strong priming when it was clearly perceived (100-ms ISI) and at both 12.5- and 25-ms ISI elicited priming for participants who did perceive the prime (**Figure 3**). As such, there were general priming effects which were not evident only for subliminal participants. Second, as discussed above, the intermediate signal strength used here led to a wide range of *d′* values, which is necessary when using regression analysis to evaluate subliminal priming. Previous studies that have restricted the range of *d′* values may thus have overestimated subliminal effects (Dosher, 1998; Miller, 2000; Sand and Nilsson, under review). Third, it might be that the stimuli used here allowed for better estimation of prime perception, *d′*. In previous studies (e.g., Finkbeiner, 2011; Muscarella et al., 2013), the prime and target have been perceptually similar (i.e., both words) so the target could interfere with the prime response, which would lead to underestimated prime perception, *d′* (Vermeiren and Cleeremans, 2012). In contrast, in the current experiment, primes and targets were highly dissimilar (i.e., a word and colored rectangle); as discussed by Van den Bussche et al. (2013), this makes it easier for participants to judge whether or not they perceived the prime. Future studies should more thoroughly investigate the possibility of the underestimation of *d′* based on prime target similarity.

The present data do not support subliminal semantic priming but is not inconsistent with the existence of subliminal semantic priming as an actual phenomenon. A single study cannot settle whether or not subliminal semantic priming is possible; doing that would require an overall assessment of the literature. It is important that such an assessment include both studies that find subliminal effects and studies that do not, as including only studies with positive results overestimates effect sizes (i.e., file drawer bias). This is especially important in this topic as many studies report small effect sizes for subliminal priming (Van den Bussche et al., 2009, 2013; Fisk and Haase, 2011; Haase and Fisk, 2011).

## Conclusion

I conclude that, at specific ISIs, both trial-to-trial and inter-individual variability in prime perception strongly influence priming effects even when signal strength is constant. This implies that priming effects measured as means across trials may arise from trials with varying degrees of perception (Van den Bussche et al., 2013; Haase and Fisk, 2015) and that priming effects measured as means across participants may arise from participants’ having varying degrees of perception (Haase and Fisk, 2015). I therefore agree with Haase and Fisk (2015) that, in the context of subliminal processing, drawing conclusions based on data averaged across trials and participants may be unwarranted as the average may be based on trials and participants with various levels of prime perception.

## Author Contribution

The author confirms being the sole contributor of this work and approved it for publication.

## Conflict of Interest Statement

The author declares that the research was conducted in the absence of any commercial or financial relationships that could be construed as a potential conflict of interest.

## Footnotes

1 Throughout this paper I use “perception” as synonymous with “conscious perception,” “subliminal” to refer to stimulus intensities below the threshold of perception, and “subliminal processing” to refer to processing of a stimulus with intensity below the threshold of perception, i.e., a process occurring in the absence of perception.

2 The actual prime stimuli were the Swedish words “röd,” “blå,” “grön,” and “gul.”
